# Pertussis Toxin Ameliorates Microglial Activation Associated With Ischemic Stroke

**DOI:** 10.3389/fncel.2020.00152

**Published:** 2020-06-26

**Authors:** Feihui Zhou, Rong Liu, Pengcheng Han, Xingkui Zhang, Zhigao Li, Shen Zhang, Chang Liu, Yang Xia, Zhiwei Tang

**Affiliations:** ^1^Department of Neurosurgery, The First Affiliated Hospital of Kunming Medical University, Kunming, China; ^2^Key Laboratory of Animal Models and Human Disease Mechanisms of Chinese Academy of Sciences, Kunming Institute of Zoology, Chinese Academy of Sciences, Kunming, China; ^3^Department of Pathology and Laboratory Medicine, Medical University of South Carolina, Charleston, SC, United States

**Keywords:** pertussis toxin, stroke, microglia, neuroprotection, neuroinflammation

## Abstract

**Objective**: To investigate the effect and the underlying mechanism of Pertussis toxin (PTX) on microglia in the setting of cerebral ischemia.

**Methods**: We tested the effect of PTX 400 ng/days on middle cerebral artery occlusion stroke model by evaluating the neurologic function, infarct size, microglial distribution, and activation. In parallel, we also tested the effect of PTX on primary cultured microglia by evaluating microglial proliferation, activation, cytokine release, and CX3CR1 expression.

**Results**: PTX reduced the poststroke infarct size, improved the neurologic function as evaluated by Longa score, and reduced microglial aggregation and activation in the infarcted area. Further, PTX significantly decreased lipopolysaccharide-stimulated microglial proliferation, the release of interleukin 1β (IL-1β) and tumor necrosis factor α (TNF-α), and the expression of CX3CR1.

**Interpretation**: PTX treatment in stroke reduced microglial accumulation and activation in the infarct zone, resulting in a better functional outcome. The benefits of PTX treatment may be attributed to the reduced production of proinflammatory cytokine such as IL-1β and TNF-α and reduced expression of chemokine CX3CR1.

## Introduction

Stroke is the second leading cause of mortality worldwide and has overtaken heart disease and become the primary cause of death in east Asian population (Moskowitz et al., 2010; Liu et al., 2011). Ischemic stroke constitutes 85% of all stroke incidences; the increasing morbidity and mortality associated with stroke pose a huge socioeconomic burden to aging society worldwide (Di Carlo, 2009). It remains a huge challenge to establish an effective therapy, but incessant efforts to search for better therapeutic approach are clinically demanding and highly valuable.

Inflammatory reactions inevitably follow a stroke event due to the hypoxia-related cell damage and tissue necrosis. The natural pathologic course starts from aseptic inflammation involving a wide spectrum of inflammatory factors. Microglial activation is a crucial step in the pathogenesis of aseptic inflammation (Xiong et al., 2016; Zhang et al., 2017).

Microglia are perhaps the most important intrinsic cerebral immune cells, constituting approximately 5% to 10% of total number of brain cells (Chan et al., 2007; Li and Barres, 2018). Microglia are the first group of immune cells that reacts to stroke event; they are activated by multiple cytokines and plasma proteins (Zhang et al., 2017). The activated microglia are key immune cells in central nervous system, as they maintain microenvironmental homeostasis by repairing the damage (Davalos et al., 2005; Kim and de Vellis, 2005). Microglia aggregate toward necrotic tissue and their peripheral regions. The accumulated microglia at early stage are M2 type, phagocytizing and removing cell debris, necrotic tissue, and toxic metabolites. After approximately 24 h, M2 microglia are superseded by M1 subtypes, which release additional proinflammatory cytokines and exacerbate cell damage (Perego et al., 2011; Wang et al., 2011, 2013). In this context, microglial activation in necrotic tissue might be a detrimental factor to accelerate neuronal death by spilling over inflammatory cytokines to by-standing neurons. Therefore, it would be beneficial to inhibit microglial activation after stroke onset. Suggested by several recent studies, however, microglial functions are heterogeneous, and their role in stroke is not uniform. Instead, microglia may play diverse functions, depending on the time and location during stroke pathogenesis (Hu et al., 2012; Xiong et al., 2016). The complexity and importance of microglia in stroke remain elusive and deserve in-depth study.

Pertussis toxin (PTX) is an exotoxin produced by *Bordetella pertussis*. PTX is used to prepare mouse model of experimental autoimmune encephalomyelitis (EAE; Tang et al., 2013). We previously found that low dose of PTX (50 ng/ml) may reduce neuronal calcium influx, thus minimizing neuronal damage and protecting cell viability in stroke (Tang et al., 2015). Although PTX, at least at a restricted dose, offers a direct protective effect on neuron, their potential pharmacologic mechanism in stroke protection may include additional key steps. We also found previously that PTX paradoxically attenuates inflammation in EAE model if we administer a relative low dose (Tang et al., 2013). Therefore, we hypothesize that inhibiting inflammatory components may contribute to the protective effect of PTX in experimental stroke. Specifically, we hypothesize that PTX modulates microglial function by inhibiting their activation and proliferation, thus ameliorating poststroke brain damage.

## Materials and Methods

### Transient Middle Cerebral Artery Occlusion Animal Model

All procedures are approved by the Institutional Animal Care and Use Committee of the first hospital of Kunming Medical University. All studies were conducted in accordance with the US Public Health Service’s Policy on Humane Care and Use of Laboratory Animals. The procedure for transient middle cerebral artery occlusion (tMCAO) model was described previously (Chen et al., 2005; Liesz et al., 2009; Wang et al., 2018). Briefly, 20–25 g C57BL/6 male adult mice were anesthetized with intraperitoneal injection of 1 mg ketamine and 0.5 mg xylazine dissolved in 1 ml normal saline, and the injection dose was 0.01 ml/g. Then, the animals were stabilized on the operating table with neck fully exposed, shaved and disinfected with 75% alcohol, and placed under the operating microscope. The skin is cut along the neck midline with blunt dissection of subcutaneous tissue. After exposure of the right internal carotid, external carotid, and common carotid arteries, a small incision was made in the common carotid artery, and a 6–0 surgical nylon monofilament with rounded tip entered the internal carotid artery approximately 10 mm until stopped by slight resistance. The filament was left in place for 60 min and then withdrawn for reperfusion. Throughout the procedure, body temperature was maintained at 37°C ± 0.5°C. Postoperatively, the animals were placed in an incubator with constant ambient temperature. Sham-operated control mice received the same surgical procedure without inserting a filament. They were separately housed and injected with 1 ml normal saline twice a day.

### Randomization and Treatment

After establishing tMCAO stroke model, we randomized mice into a therapy group (*n* = 12) and a placebo control group (*n* = 12). Sham group also was established at the same time including 12 mice. The therapy group was treated with PTX 400 ng/days dissolved in 1 ml normal saline, and the control group and sham group were given 1 ml normal saline intraperitoneally, which lasted for 3 days.

### Neurological Deficit Assessments (Longa Score)

Neurological deficit assessments were performed by investigators who were blinded to the experimental groups, as described previously (Tang et al., 2015; Yin et al., 2015; Xie et al., 2018). Briefly, rating scale was used as follows: score 0 was defined as the complete absence of neurological deficit; score 1 was defined as that front paws incompletely extended and mild neurological deficit; score 2 was defined as lateral turning while walking and moderate neurological deficits; score 3 was defined as lateral jumping of animal body and severe neurological deficits; score 4 was defined as lack of “conscious” response to noxious stimuli.

### Microglial Culture

The method was previously described in detail (Lian et al., 2016). Briefly, the mice born within 24 h were sacrificed by decapitation under sterilized condition. The brain tissue was placed in a 35-mm Petri dish containing an anatomical fluid and placed on ice. Tissues were dissected under a dissecting microscope; the cortex and hippocampus were preserved, and the meninges were carefully removed. The brain tissue was smashed, collected in a centrifuge tube, and placed vertically. Then the tissue was mixed gently with 0.25% trypsin and incubated in a 37°C for 10 min. After gentle trituration, the cell suspension was filtered with 70-μm cell strainer, and the supernatant was collected after centrifugation 1,600× *g* for 5 min. Cell counting was performed using a hemocytometer. The cell suspension was placed into a polylysine-coated Petri dish at approximately 5 × 10^4^/ml. The Petri dishes were placed in a 37°C incubator, and the media was changed at 24 h after inoculation and then changed 50% of the volume every 72 h until used for lipopolysaccharide (LPS; 400 ng/ml) stimulation or other experiments (PTX, 50 ng/ml).

### 2,3,5-Triphenyltetrazolium Chloride Staining

As previously described (Wang et al., 2018), the animals were quickly anesthetized, and the brains were removed rapidly and frozen at −20°C for 5 min. Coronal slices were cut anterior to posterior at 2-mm interval, and sections were immersed in 2% triphenyltetrazolium chloride (TTC; Sigma–Aldrich, St. Louis, MO, USA) at 37°C for 20 min. The presence of infarction was determined by the area that stained negative with TTC and was quantitated with ImageJ software (National Institutes of Health, Bethesda, MD, USA).

### Immunohistochemistry for *in vivo* and *in vitro* Microglia

Seventy-two hours after MCAO, terminally anesthetized mice were perfused intracardially with saline followed by 4% paraformaldehyde. The fixed brains were embedded in paraffin and cut into serial 5-μm-thick coronal slices. Immunohistochemistry was performed with antibodies against IBA-1 (Fujifilm-Wako, Tokyo, Japan) to identify microglia, anti-CX3CR1 antibody (Invitrogen, Carlsbad, CA, USA) to identify Chemokine receptor, and anti-Ki67 antibody (#9027; Cell Signaling, Danvers, MA, USA) to identify proliferative cells. The secondary antibodies we used were goat anti-mouse immunoglobulin G (IgG; cat. #401215; Millipore, Burlington, MA, USA) and goat anti-rabbit IgG (cat. #AP307P, Lot #2899737; Millipore, Burlington, MA, USA).

For cultured microglia, the cells were fixed in 4% paraformaldehyde, treated with 0.3% Triton X-100 for 20 min, washed twice with phosphate-buffered saline, and stained with antibodies against IBA-1 to identify microglia and CX3CR1 to identify chemokine receptor. The nuclear counter stain was performed using 0.5 μg/ml DAPI. Results are presented as IBA-1^+^ or CX3CR1^+^ cells per images of 20× magnification field images.

### Microglial Proliferation Assay

We used two independent assays to assess microglial cell numbers after LPS stimulation and compared them with PTX-treated cells. MTT assay was performed using Vybrant MTT Cell Proliferation Assay Kit (V13154; Thermo Fisher Scientific, Waltham, MA, USA) according to the manufacturer instruction. Briefly, cultured primary cells were divided into various treatment group, control group, and blank group (see “Results” section). Five percent MTT solution is added and incubated at 37 degrees for 2 h. After removing medium and adding dimethyl sulfoxide with gentle shaking, we measured the absorbance at 570 nm.

Sulforhodamine B (SRB) assay was performed using CYTOSCAN^TM^ SRB Assay kit (cat. 786213; G-Biosciences, St. Louis, MO, USA) according to the manufacturer instruction. Briefly, microglia were plated in 48-well plates at densities varying from 1 × 10^4^ to 4 × 10^4^ cells/well. One day after plating, the cells were treated with vehicle control, LPS, LPS + PTX, and PTX separately at indicated dosage for another 48 h. The cells were then fixed with 10% trichloroacetic acid followed by staining with 0.4% (wt/vol) SRB for 30 min at room temperature, and extra dye was washed with 1% acetic acid. Finally, 10 mM Tris base was added to dissolve the dye, and the optical densities at 530 nm were measured using a spectrophotometric plate reader (Bio-Tek, USA). Data were expressed as percentage of viable cells compared to the control culture.

### Western Blot Analysis

The brain tissue of the affected side of MCAO mice was crushed in liquid nitrogen, and the tissue was placed on lysate (RIPA + 1% protein inhibiting enzyme) to be lysed on ice. After centrifugation, the supernatant was used for Western blot analysis. Anti–IBA-1 (1:500), anti-CX3CR1 (1:500), and anti–β-actin (1:1,000) were used as primary antibodies. Protein detection and quantification were performed according to the manufacturer’s protocol.

### Enzyme-Linked Immunosorbent Assay

Microglial cultured media were harvested to measure interleukin 1β (IL-1β) and tumor necrosis factor α (TNF-α) using Quantikine enzyme-linked immunosorbent assay (ELISA) kit (cat. number MTA00B for TNF-α, cat. MLB00C for IL-1β; R&D System, Minneapolis, MN, USA). Briefly, specimen was measured in parallel with a series of standard diluted control sample, which was used to establish standard curve. The optical density of each well was measured using a microplate reader set to 450 nm. Wavelength correction was performed at 540 and 570 nm.

### Statistical Analysis

The results are presented as the means ± SEM. Statistical differences between two groups were evaluated by the two-tailed unpaired Student’s *t*-test. Multiple comparisons were performed with two-way analysis of variance (ANOVA) accompanied by Bonferroni *post hoc* test. *P* < 0.05 was considered significant.

## Results

### PTX Reduced Poststroke Infarct Volume and Functional Deficit

**Figure 1:**
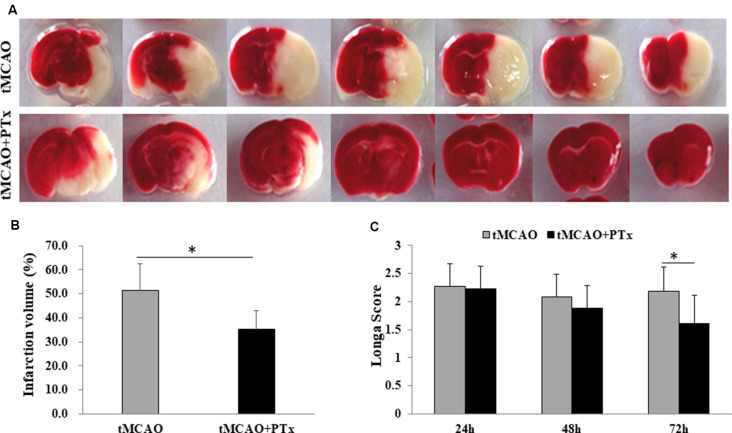
Pertussis toxin (PTX) reduced poststroke infarct volume and functional deficit. **(A)** Representative images of triphenyltetrazolium chloride (TTC)-stained brain slice in stroke mice with or without PTX treatment. The infarction area (non-stained region) was quantified in each slice and cumulated as a percentage of total volume. Note PTX-treated animals showed relative less area of infarction. **(B)** Statistical comparison of infarction volume between stroke mice with or without PTX treatment. **(C)** Neurological deficit was assessed using Longa score (*P < 0.05).

Stroke animals in PTX treatment group received daily intra-abdominal injection of PTX (400 ng per day, 1 ml) for three consecutive days. Stroke animals in the control group received daily intra-abdominal injection of 1 ml saline. The animals were sacrificed 72 h poststroke, and the brain slices were stained with TTC to evaluate infarct volume. PTX therapy significantly reduced infarct volume of therapy group (average infarct percentage = 34% ± 8%) compared to that of the saline-treated group (52% ± 12%, *P* < 0.05, Student’s *t*-test), suggesting PTX therapy effectively reduces cell death associated with MCAO-induced ischemia (Figures 1A,B). Further, Longa scores were assessed at 24, 48, and72 h poststroke. At 24 and 48 h poststroke, the average Longa score in the therapy group was not different from that of the saline-treated group. At 72 h poststroke, however, the average score was 1.6 in the therapy group, significantly reduced compared to 2.3 in the control group (*P* < 0.05), suggesting that 3 days’ treatment with PTX prevented functional deficit (Figure 1C). The lack of therapeutic effect at early stage is perhaps due to insufficient time for the neurological dysfunction to be fully developed. These data are consistent with our previous study, supporting the hypothesis that PTX therapy reduces necrosis and functional deficit (Tang et al., 2015).

### PTX Reduced Microglial Quantity After MCAO-Induced Ischemia

**Figure 2:**
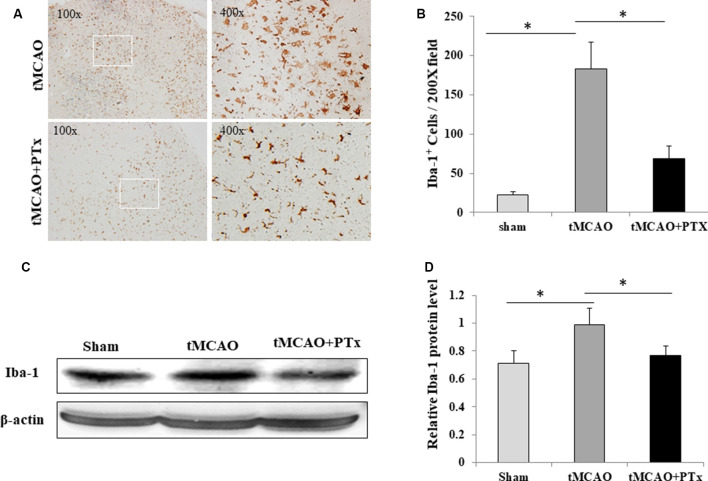
PTX reduced microglial quantity after MCAO-induced ischemia. **(A)** Representative images of IBA-1–stained microglia in brain sections obtained from transient middle cerebral artery occlusion (tMCAO) mice. **(B)** Statistical comparison of IBA-1^+^ cell counts between MCAO mice treated with or without PTX. **(C,D)** The ratio of IBA-1/β-actin immunoblot density was compared between tMCAO mice with or without PTX treatment (*P < 0.05).

We evaluate microglial aggregation in MCAO ischemic brain area by counting IBA-1–positive cells. At 72 h poststroke, massive IBA-1–positive cells accumulated in necrotic regions, and their morphology transformed from resting state into enlarged, amoeba-like cell body. The quantity of IBA-1–positive microglia was reduced in the PTX-treated group, and they presented as branch-like morphology, a shape in between the activated and resting states (Figure 2A). The IBA-1–positive cell count was 25 ± 4 per 200× microscopic field in non-stroke (sham surgery) mice brain. The average count was 183 ± 46 at 72 h poststroke in saline-treated stroke mice, but was as low as 63 ± 22 in PTX-treated stroke mice (*P* < 0.05, one-way ANOVA with *post hoc* Bonferroni test; Figure 2B). Consistent with cell count, the IBA1 expression level was significantly reduced by 21% in PTX-treated mice, as compared to the saline-treated MCAO group (*P* < 0.05, one-way ANOVA with *post hoc* Bonferroni test; Figures 2C,D).

### PTX Reduced Microglial Proliferation *in vivo* and *in vitro*

**Figure 3:**
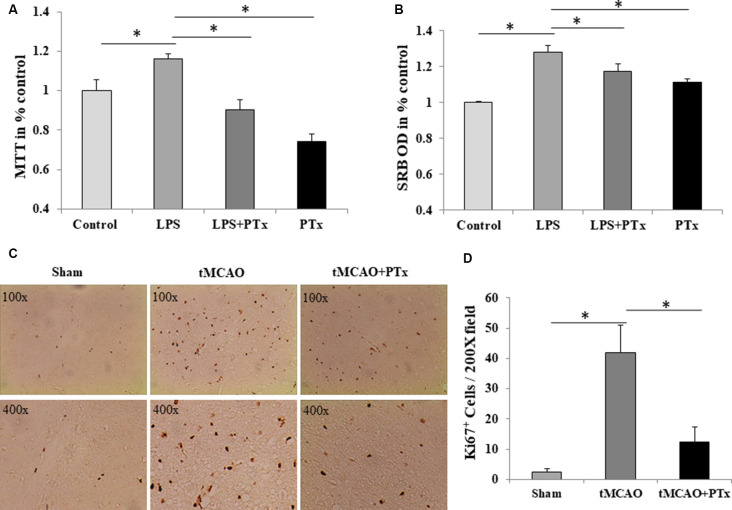
PTX reduced microglial proliferation *in vivo* and *in vitro*. **(A)** PTX treatment (50 ng/ml) reduced MTT quantity compared to lipopolysaccharide (LPS) stimulated microglia. **(B)** PTX treatment (50 ng/ml) reduced Sulforhodamine B (SRB) quantity compared to LPS stimulated microglia (*P < 0.05). **(C)** Representative images of Ki67^+^-stained cells in brain sections obtained from tMCAO mice. **(D)** Statistical comparison of Ki67^+^ cell counts between MCAO mice treated with or without PTX (*P < 0.05).

The source of microglia in stroke may come from blood-borne macrophages that enter central nervous system through damaged blood–brain barrier. Alternatively, resident microglia in brain may rapidly proliferate. As IBA-1 is a specific marker for microglia, it is likely that PTX reduced microglial cell number by inhibiting their proliferation. To evaluate intrinsic microglial proliferative capability, first we count the Ki67-positive cells. At 72 h poststroke, massive Ki67-positive cells was observed in necrotic regions, and PTX treatment could reduce Ki67-positive cells (Figure 3C). The Ki67-positive cell count was 3 ± 2 per 200× microscopic field in nonstroke (sham surgery) mice brain. The average count was 42 ± 9 at 72 h poststroke in saline-treated stroke mice, but was as low as 12 ± 5 in PTX-treated stroke mice (*P* < 0.05, one-way ANOVA with *post hoc* Bonferroni test; Figure 3D). Then, we stimulated primary cultured microglia with LPS, mimicking the pathophysiologic process *in vivo* (Lund et al., 2006). Lipopolysaccharide significantly increased microglial proliferation resulting in a larger population of surviving cells that is 20% higher than non-stimulated microglia. PTX inhibited LPS-stimulated microglial proliferation by approximately 30% (Figure 3A). Consistently, SRB assay revealed a similar suppression of LPS-stimulated microglia (Figure 3B).

### PTX Reduced Microglial Cytokine Release in Response to LPS Stimulation

**Figure 4:**
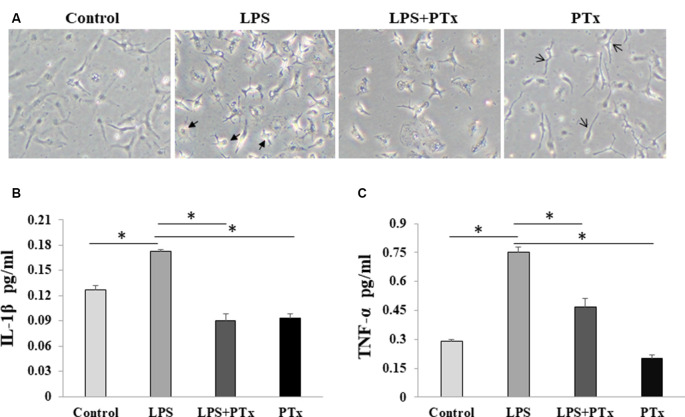
PTX reduced microglial cytokine release in response to LPS stimulation. **(A)** Representative images of cultured microglia at the time of harvest at 24 h. Two kinds of arrows indicate the distinct large amoeboid (thick arrow symbol) and long spread body cells (thin arrow symbol) and long spread body cells. **(B)** ELISA quantification of IL-1β in conditioned media. **(C)** ELISA quantification of TNF-α in conditioned media (*P < 0.05).

We evaluate microglial cytokine release by measuring representative inflammatory factors, IL-1β and TNF-α, using ELISA assay. Cultured microglia were added with 50 ng/ml PTX (treatment group), LPS only, LPS + PTX, or with equal volume culture media (control group). The cultured cells and media were harvested at 24 h after LPS stimulation. When the cultured cells were treated with PTX only without LPS stimulation, the microglia showed a similar morphology as control, suggesting that PTX, at least at this concentration of 50 ng/ml, is not toxic to microglia. Lipopolysaccharide-stimulated microglia morphed into a distinct large amoeboid cell, whereas microglia treated with PTX demonstrated morphology similar to resting microglia with long spread cell body and scant branches (Figure 4A). We harvest the media to measure IL-1β and TNF-α in the conditioned media. In LPS-stimulated media, IL-1β was increased from 0.128 ± 0.003 to 0.165 ± 0.001 pg/ml, and TNF-α was increased from 0.128 ± 0.001 to 0.75 ± 0.003 pg/ml. In the PTX treatment group, IL-1β was reduced from 0.165 ± 0.001 to 0.091 ± 0.0004 pg/ml, and TNF-α was reduced from 0.75 ± 0.003 to 0.46 ± 0.005 pg/ml (Figures 4B,C; *P* < 0.05, one-way ANOVA with *post hoc* Bonferroni test).

### PTX Reduced Expression of CX3CCR1 by Microglia

**Figure 5:**
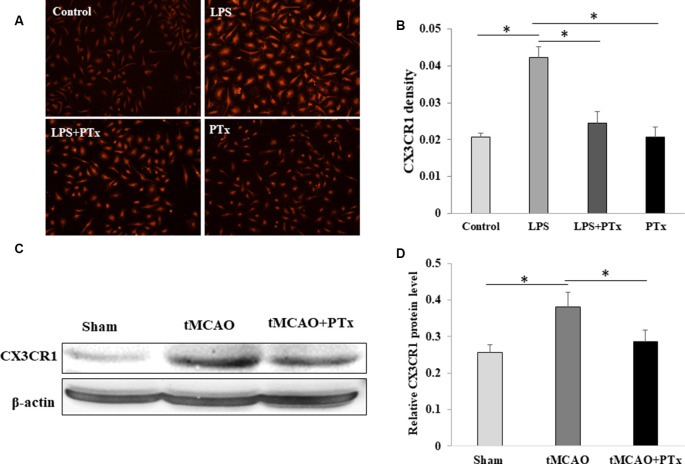
PTX reduced expression of CX3CCR1 by microglia. **(A,B)** Representative images **(A)** and statistical comparison **(B)** of fluorescent density of CX3CR1-binding antibody in cultured microglia. Note PTX decreased CX3CR1 fluorescent density. **(C,D)** The density ratio of CX3CR1/β-actin was decreased in PTX treated stroke mice (*P < 0.05).

In primary cultured microglia without LPS stimulation, CX3CR1 expression is low with the average fluorescent intensity of 0.02 ± 0.001. Lipopolysaccharide-stimulated microglia demonstrated significant increase in CX3CR1 fluorescent intensity up to 0.043 ± 0.003. PTX treatment of LPS-stimulated microglia reduced CX3CR1 fluorescent intensity down to 0.025 ± 0.003. PTX treatment alone without LPS stimulation was similar to control (Figures 5A,B). Consistent with cultured microglia *in vitro*, CX3CR1 expression in MCAO stroke mouse was increased 30% as compared to non-stroke sham-surgical mice. PTX–treated stroke mice decreased the CX3CR1 expression back to a level similar to sham-surgical mice (Figures 5C,D).

## Discussion

The primary goal for modern stroke therapy is to salvage tissue in ischemic penumbra aiming to minimize long-term functional deficit. Since the proposal of penumbra concept in 1970s, it is still the primary therapeutic goal as of today (Campbell et al., 2014; Davis and Donnan, 2014; Baron, 2018). While early reperfusion by thrombolysis or thrombectomy is a crucial step, reducing the cell vulnerability is equally essential to minimize reperfusion injury. The infarct tissue inevitably induces inflammation by activating microglia, the resident scavenging cells in the central nervous system. While microglia play an important role in phagocytosing the necrotic tissue, they release multiple proinflammatory factors to recruit lymphocytes and neutrophils (Neumann et al., 2018). The large amount of proinflammatory cytokines may damage the neighboring neurons in the penumbra. Therefore, fine-tuning the microglial reaction after stroke is a potential therapeutic target in tandem with neuroprotection, and recent studies provide encouraging progress (Bok et al., 2017).

In this study, we showed that PTX decreased infarct volume in MCAO stroke model at 72 h poststroke, a timeline consistent with cerebral perfusion kinetics(Tang et al., 2015). In this our study, we focused on the acute stage of ischemic stroke; thus, we investigated the effects of PTX on stroke at 24 and 72 h after treatment. While the PTX did not reduce the infarct volume and improve the neurological function at 24 h after stroke, and the IBA-1^+^ cells increased only mildly, we did not observe that PTX can inhibit microglia activation at this time point, whereas at 72 h, PTX inhibited microglia activation and decreased infarct volume dramatically. In different disease, the time point that PTX inhibits inflammatory responses is different (Andreasen and Carbonetti, 2008; Connelly et al., 2012). PTX also protected neuronal apoptosis; the underlying mechanisms include amelioration of calcium overload (Tang et al., 2015). However, given the potent effect of PTX, a direct neuroprotection is unlikely the only mechanism. PTX may have even broader effects on multiple players in the context of ischemic stroke. Therefore, we tested the effects of PTX on microglial activation in this study.

Microglial proliferation was observed in our tMCAO model as indicated by increased IBA-1^+^ and Ki67^+^ cells. PTX effectively reduced the number of microglia; the reason should include both limiting the recruitment of microglia and inhibiting the proliferation of microglia. Several articles have reported that PTX could inhibit microglia migration (Yin et al., 2010). In our study, we focus on the inhibition of microglia proliferation by PTX. We found cell proliferation increased remarkably 72 h after stroke, whereas PTX can inhibit this proliferation. This *in vivo* effect was modeled *in vitro* by stimulating cultured microglia with LPS but suppressed by PTX. The cultured microglia were assessed by two independent methods: MTT and SRB assays. Sulforhodamine B measures the level of constitutive protein corresponding to the amount of live cell because dead cells were washed away prior to the assay. MTT assay is predicated not only on cell number but also on mitochondrial quantity. Therefore, the suppressive effect of PTX appears larger in MTT assay than that in SRB assay. Nevertheless, both assays confirmed the inhibitory effects of PTX on LPS-stimulated microglial proliferation.

PTX could change microglia morphology. The authors showed that PTX induced a dose-dependent morphological transformation. At 125 pg/ml, PTX almost does not change microglial morphology. Typical change into small and rounded macrophages happened at 125 ng/ml; at 12.5 mg/ml, many cells showed lamellar growth surfaces and short filopodial processes indicative of amoeboid morphology (Kalla et al., 2003). In our study, we use 50 ng/ml of PTX to treat the culture microglia, which did not change the cells into small and rounded macrophages; the microglia showed a similar morphology as control, suggesting that PTX, at least at this concentration of 50 ng/ml, is not toxic to microglia, and it can inhibit the LPS effect to microglia. In another article (Ahmad et al., 2019), at 2.75 ng/ml, PTX can inhibit maturation of human monocyte precursors into the more phagocytic macrophage cells. It is similar to our data. All these indicated that low concentration of PTX is not toxic to microglia, and it can inhibit microglia change to macrophages.

Upon microglial activation, multiple cytokines are released from microglia. Among these, IL-1β is the immediate early response cytokine (Kim et al., 2006), which further orchestrates underlying inflammatory response (Kaushik et al., 2013). TNF-α is a signal linking inflammation and excitotoxicity (Olmos and Lladó, 2014). Activated microglia secrete TNF-α, which further stimulates additional microglia *via* autocrine mechanism (Kuno et al., 2005). Meanwhile, TNF-α has a direct effect on glutamate receptor inducing unabated calcium influx resulting in excitotoxicity (Han and Whelan, 2010). Therefore, we chose to analyze microglia-secreted IL-1β and TNF-α as the most relevant and representative cytokines in our study, because IL-1β is an early marker for microglial activation, and TNF-α is an effector cytokine for neuronal damage. Our data showed that PTX reduced LPS-stimulated IL-1β and TNF-α secretion. Although LPS and their targeted TLR are not known to be associated with G-protein signaling, secondary signaling (e.g., purinergic receptor signal) may be subject to G-protein–related modulation (Netea et al., 2009), hence a potential step that can be blocked by PTX.

The chemokine fractalkine (CX3CL1)/CX3C chemokine receptor 1 (CX3CR1) system is a critical signaling system modulating the communication between neurons and microglia (Harrison et al., 1998; Mizuno et al., 2003). CX3C chemokine receptor 1 is expressed predominantly in astrocytes and microglia (Maciejewski-Lenoir et al., 1999), but moderately in neurons as well (Meucci et al., 2000; Wang et al., 2018). Our previous work demonstrated that CX3CR1-mediated microglia activation was associated with neuronal apoptosis in the penumbra 72 h after transient cerebral artery occlusion in mice, and CX3CR1 deficiency suppresses the neurotoxic effect of microglia (Tang et al., 2014). Therefore, CX3CR1 upregulation is a necessary event in microglia-mediated neurotoxicity. Here, we show the suppressive effects of PTX on microglial CX3CR1 expression *in vitro* and *in vivo*, suggesting that CX3CL1–CX3CR1 is a likely target of PTX. In fact, CX3CR1 belongs to a family of G-protein–coupled receptor functionally interacting with Gαi, a target of PTX (Poniatowski et al., 2017). Further study is needed to elucidate the mechanism why blocking a downstream signaling protein provides a negative feedback to upstream receptor and reduced the expression level of CX3CR1.

The mechanism that PTX inhibits microglia remains elusive. Nevertheless, it has been noted that PTX suppresses the function of pulmonary alveolar macrophage (Carbonetti et al., 2007), perhaps as a strategy to fend host innate immune system and establish *B. pertussis* infection. PTX targeted several G-protein signaling pathways in macrophage (Lattin et al., 2007; Carbonetti, 2010). Given that microglia and macrophage are derived from common embryonic cell lineage and share common molecular mechanisms in their functions (Li and Barres, 2018), it is not surprising to observe that PTX inhibits microglial proliferation and inflammatory response. Indeed, microglial inhibition by PTX was also observed in EAE; the total number of IBA-1–positive cells was reduced by 69.6% with PTX treatment (Yin et al., 2014).

The suppression of microglial activation by PTX is in tandem with a direct neuroprotective effect of PTX. We previously demonstrated that PTX significantly reduced calcium influx *via* glutamate receptor and voltage-gated calcium channel (Tang et al., 2015). Further, our recent data suggest that neuronal CX3CR1 upregulation may be directly related to glutamate receptor–mediated calcium influx (Tang et al., 2014). Therefore, it is possible that PTX blocks CX3CR1 signaling in neurons, hence indirectly reduced calcium overload. One potential possibility is that reduced cell death decreases microglial activation; hence, the suppressive effect of PTX on microglia may be attributed to an indirect outcome subsequent to better neuronal protection. While this indirect mechanism certainly exists, PTX likely exerts direct effect on microglia; as shown in this study, primary cultured microglia *in vitro* were equally suppressed by PTX as well as *in vivo*. Therefore, PTX provides a neuroprotective effect through multiple nonexclusive mechanisms involving both neurons and microglia.

## Conclusion

We demonstrated here that PTX ameliorated postischemic microglial proliferation, activation, cytokine release, and the expression of proinflammatory signal molecules. The suppression of microglia in alignment with direct neuroprotection suggests that PTX has a multifaceted therapeutic function, a unique advantage in treating ischemic stroke.

## Data Availability Statement

All datasets generated for this study are included in the article.

## Ethics Statement

The animal study was reviewed and approved by the Institutional Animal Care and Use Committee of the first hospital of Kunming Medical University.

## Author Contributions

FZ is the first author, she is in charge of most of the experimental works and writing. ZT is the corresponding author, he is in charge of project funding application, project design and writing. RL is in charge of experimental guidance and project design. PH is in charge of article modification. XZ and ZL are in charge of making animal model. SZ and CL are in charge of immunohistochemistry and TTC. YX is in charge of ELISA.

## Conflict of Interest

The authors declare that the research was conducted in the absence of any commercial or financial relationships that could be construed as a potential conflict of interest.
